# Advances in Quantum-Dot-Based Displays

**DOI:** 10.3390/nano10071327

**Published:** 2020-07-06

**Authors:** Yu-Ming Huang, Konthoujam James Singh, An-Chen Liu, Chien-Chung Lin, Zhong Chen, Kai Wang, Yue Lin, Zhaojun Liu, Tingzhu Wu, Hao-Chung Kuo

**Affiliations:** 1 Department of Photonics & Graduate Institute of Electro-Optical Engineering, College of Electrical and Computer Engineering, National Chiao Tung University, Hsinchu 30010, Taiwan; s101328035@gmail.com (Y.-M.H.); jamesk231996@gmail.com (K.J.S.); arsen.liou@gmail.com (A.-C.L.); hckuo@faculty.nctu.edu.tw (H.-C.K.); 2 Institute of Photonic System, National Chiao Tung University, Tainan 71150, Taiwan; 3 Department of Electronic Science, Fujian Engineering Research Center for Solid-State Lighting, Xiamen University, Xiamen 361005, China; chenz@xmu.edu.cn (Z.C.); yue.lin@xmu.edu.cn (Y.L.); 4 Department of Electrical and Electronic Engineering, Southern University of Science and Technology, 1088 Xueyuan Blvd, Xili, Nanshan, Shenzhen 518055, China; wangk@sustc.edu.cn (K.W.); liuzj@sustech.edu.cn (Z.L.)

**Keywords:** quantum dots, light-emitting diodes (LEDs), white LEDs, high efficiency, high polarization, perovskite

## Abstract

In terms of their use in displays, quantum dots (QDs) exhibit several advantages, including high illumination efficiency and color rendering, low-cost, and capacity for mass production. Furthermore, they are environmentally friendly. Excellent luminescence and charge transport properties of QDs led to their application in QD-based light-emitting diodes (LEDs), which have attracted considerable attention in display and solid-state lighting applications. In this review, we discuss the applications of QDs which are used on color conversion filter that exhibit high efficiency in white LEDs, full-color micro-LED devices, and liquid-type structure devices, among others. Furthermore, we discuss different QD printing processes and coating methods to achieve the full-color micro-LED. With the rise in popularity of wearable and see-through red, green, and blue (RGB) full-color displays, the flexible substrate is considered as a good potential candidate. The anisotropic conductive film method provides a small controllable linewidth of electrically conductive particles. Finally, we discuss the advanced application for flexible full-color and highly efficient QD micro-LEDs. The general conclusion of this study also involves the demand for a more straightforward QD deposition technique, whose breakthrough is expected.

## 1. Introduction

Quantum dots (QDs) are miniscule semiconductor particles, whose sizes are on the order of a few nanometers. They exhibit unique electronic and optical properties that are different from those of the bulk semiconductor materials. QDs have discrete electronic states, like those of natural atoms, and their electronic wave function is somewhat analogous to that of a real atom. Hence, they are frequently referred to as artificial atoms [1,2,3]. Because of their distinctive properties, QDs have found applications in a variety of modern day technologies including solar cells [4,5,6,7,8], photodetectors [9], photodiodes [10], field-effect transistors [11], biological systems [12,13,14,15], and light-emitting diodes [16,17,18].

QDs are used as color down-converters for light-emitting diodes (LEDs) to accomplish efficient illumination sources and high-quality displays. Both electrically and optically pumped quantum dots are used with LEDs [19]. Along with the radiative energy transfer from LEDs to QDs, another mechanism involves Forster resonance energy transfer (FRET), which is responsible for the transfer of non-radiative energy from LEDs to QDs. FRET, also referred to as non-radiative resonant energy transfer (NRET) is sufficiently strong to be observed when emissive quantum wells of the LED are in close contact with the QD phosphor layer. Researchers made use of innovative ideas to build QD-LEDs with better performances. The availability of multiple material choices for QDs indicates their variability across different reports. Displays based on QD electroluminescence (EL) have higher capability than either of the QD PL-based OLED or liquid crystal technologies to provide the best solution of wide gamut and pure black color. The QD EL display (“QD-LED display” and also called “QLED” or “EL-QLED” display) has electrons and holes pumped into QDs where they recombine to directly produce photons for the main red, green, or blue [20]. However, in this review, we do not categorize QDs on the basis of their material or design, as this deducts from the prime focus of our discussion, i.e., the joint use of optically excited QDs and LEDs to accomplish QD-LEDs. Illumination sources and display devices based on QD-LEDs demonstrated in the past few years are discussed separately in the following sections. Energy is transferred from active LED to QDs by radiative energy transfer and NRET. In direct radiative energy transfer, the absorption spectrum of acceptors, i.e., QDs, must correspond to the emission spectrum of donors, i.e., the active LED. In contrast, in NRET, donors and acceptors must be in close contact [21]. Both of these energy transfer mechanisms can generate electron-hole pairs in QDs and hence generate radiation. An efficient QD-LED device must be designed such that both of these mechanisms can contribute to the process of energy transfer with minimum losses. QDs absorbing ultraviolet (UV)/blue wavelengths and emitting blue, green, and red colors are used with a UV or blue LED for the realization of QD-LEDs.

Recent QD-LED televisions with an edge-lit lenses promoted the first solution to QDs in consumer displays. It used a dispersion of QDs in a polymer embedded in a glass tube placed at the edges of a screen, over a strip of LEDs. This technique has many disadvantages including decreased temperature production. It proved challenging for early QDs that suffered from thermal instability issues; including a very heavy hermetic tube to ensure continued reliability of operation. The current technology of choice for QD-LCD televisions is the QD films in displays. First, quantum dots based on cadmium selenide or indium phosphide were overlaid on a blue LED backlight and incorporated into prototypical LCD matrices, which provided the benefits of QD’s color performance. Because of its higher performance with luminous efficiency and wide-gamut, CdSe-based quantum dots are normally used in the past few years. However, the Cd has the negative environmental effects. Consequently, QDs for InP and perovskite are considered candidates for solving the problem. The latest industry standard solution for producing QDs on screens is cadmium-free films. Yet there is still a consensus that QD color filters (QDCFs) of the next generation are still an upcoming stop on the QD research and development route.

Among various types of QDs, such as CdSe and InP, perovskite quantum dots (PQDs) exhibit several remarkable optical characteristics, including high photoluminescence quantum yield, tunable emission wavelength, high color purity, making them a possible candidate for next-generation cost-effective display technology [22,23,24,25]. Consequently, PQDs have been sought after in the field of research over the past few decades owing to their quantum confinement effect and resistance to various defects. Several synthetic routes were attempted to find a method that can reliably manufacture stable and defect-resistant perovskite quantum dots. Two major routes have now been developed for the synthesis: room-temperature synthesis and hot injection synthesis. The first one involves mixing cesium halide (CsX) and lead halide (CsX_2_) into a good solvent such as dimethyl sulfoxide (DMSO) or N,N-dimethylformamide (DMF) followed by the addition of capping ligands like oleic acid and oleylamine with vigorous stirring. The mixture is then added to a vigorously stirring flask containing a poorer solvent such as toluene, so that the perovskite quantum dots begin to precipitate and can be further separated by centrifugation. The second method is the so-called “hot-injection” method involving the preparation of cesium oleate in 1-octadecene (ODE) under argon at 150 °C by stirring of cesium carbonate and oleic acid. Lead halide is separately dried in ODE by vacuum heating, and capping ligands including oleic acid and oleylamine are added under argon to dissolve it completely. Then the solution of cesium oleate is stirred for 5–10 s at 150 °C before it gets precipitated which is further separated by centrifugation. Compared to CdSe- and InP-dependent QDs, PQDs have several advantages, such as tunability of emission wavelengths from blue to red, narrow full width at half maximum (FWHM), facile fabrication, etc., [26]. However, several problems impede the execution of PQDs in terms of presenting them as a color converter for display applications, and some of the challenges are addressed in this review. The optical properties of PQDs may be easily impaired by different environmental influences, such as heat, moisture, high-energy radiation, etc., which can alter their surface properties and stability in the long term [27]. Furthermore, the QDs configuration is important to assess their stability, as the QD composition and interaction between the atoms are determining factors for the QD optical properties and stability [27]. Numerous researchers reported that inorganic PQDs exhibit superior properties for optical display compared to other types of PQDs in terms of low PL quenching and PL peak shifting at elevated temperature, as shown by Sinatra et al. [27,28]. While PQDs have excellent optical properties, their display applications are limited to green emitting CsPbBr_3_ PQDs. Moreover, they are not appropriate for display applications as they suffer from high thermal quenching at higher temperatures, leading to stability issues.

Koscher et al. and Azpiroz et al. showed that the causes behind this issue of the PQDs are surface and bulk defects that can induce surface traps and ion migration, respectively [29,30]. Hence, these problems need to be overcome such that PQDs can be successfully introduced for display applications without stability issues. Numerous researchers explored various approaches to resolve these stability issues. Sinatra et al. have suggested three approaches to enhance PQDs reliability for display applications. The first is to reduce PQDs defects, in particular halide vacancies, through improving the synthesis condition by insertion of additional halide in the system [31]. The second is the treatment of PQDs with strong ligands. In most PQDs, oleic acid and oleylamine act as ligands for PQDs synthesis, and they are likely to be detached from the surface because of their weak binding to the surface at higher temperatures, leading to surface defects. The third is to provide protection for PQDs in a post-treatment method by supplying thin inorganic oxide precursors. Wei et al. suggested several ways of improving the stability of PQDs that are very similar to those described above, such as compositional engineering, surface engineering, matrix encapsulation, and device encapsulation [32]. Another explanation for this instability is the movement of protons between oleic acid (OA) and oleylamine (OLA), which induces significant ligand loss. Addressing this issue, Cai et al. reported on the enhancement of stability by suppressing the inter-ligand proton transfer and applying polystyrene coating. This was achieved by replacing oleylamine (OLA) with cetyl trimethylammonium bromide, which cannot be protonated, thereby suppressing the transfer of protons between the ligands and improving the stability in PQDs. Further, to improve moisture and thermal stability, PQDs can be further composited with carboxyl-functionalized polystyrene (cPS) via chemical interactions. Further, Lv et al. proposed multiple encapsulation techniques to improve the stability of metal halide PQDs by inhibiting light-induced decomposition and concentrating on enhancing chemical and thermal stability [33]. Several encapsulation methods include the sol-gel method, template-assisted method, physical method, and microencapsulation method which can be found in the literature. However, there is still a need for further improvement in the performance of these encapsulated PQDs to meet the increasing demand for various practical applications.

In this review, in order to enhance mass-transfer, several efficient and stable full-color LED devices are presented with various QDs patterning technologies. Inkjet-printed QDs, which combine the polymer and QD technologies, are low-cost, mask-free, and represent a simple and rapid technology. Researchers successfully demonstrated a fine linewidth (micrometer-scale) and uniform red and green QDs. Because of these excellent characteristics, QDs are relatively suitable for many applications of display technologies such as white LEDs, flexible systems containing sensors, actuators, etc. Meanwhile, PQDs are regarded as forefront material owing to their high efficiency and color purity. Nevertheless, they have encountered several challenges that can be easily overcome and this article discusses some of the solution approaches. Hence, PQDs have the potential to demonstrate lighting application and flexible display technology if the PQDs are well stored and if they can be stable enough to bear high energy radiation.

## 2. Micro-LED Display and QD Color-Conversion Technology

### 2.1. Background of Micro-LED Display

Micro-LED (µLED) display technology emerged in the recent years as a promising display technology. This technology is advocated as the ultimate choice for future displays by several researchers across the globe. The potential of µLEDs to replace conventional display technologies is owing to the combination of their self-emissive mechanism and inorganic material characteristics. The expected (or even demonstrated at the research level) better performance of µLED displays in terms of luminance, efficiency, power consumption, contrast ratio, lifetime, and response time makes them an attractive topic of research [34,35]. µLED displays are suitable for a number of applications like wearable watches, mobile phones, automotive head-up displays, AR/VR, micro projectors, and high-end televisions [36]. To use µLEDs as a display, we need to have an array of them emitting all three primary colors, i.e., red, green, and blue (RGB). Full-color µLED displays may be assembled into RGB LED matrices using mass-transfer technologies [37]. The approach using µLED chips of primary colors simultaneously to obtain full colored µLEDs comes with some crucial drawbacks. First, there is no suitable material to grow efficient green emitting LEDs due to the low efficiency emission forming a green gap among other wavelengths. Second, LEDs with different colors are usually grown on various substrates and are subject to different operating conditions. Thus, a complex circuitry is required for their integration, which is costly. These concerns along with many other significant challenges such as the low transfer yield, slow transfer time, high fabrication cost, and inspection and repair difficulties, limit the use of the above-mentioned approach to realize full-color µLEDs.

An alternative approach to realize full-color LEDs is to combine single-color LEDs and certain color-converting material, which is usually a phosphor. For LEDs, phosphor-assisted color down-conversion is a process through which a phosphor material absorbs short wavelengths and re-emits relatively longer wavelengths after energy down-conversion. In common practice, a blue or violet LED is used in combination with a broad, yellow emitting phosphor. Emitted colors are tunable by customizing the proportions of direct emissions from the LED and phosphor material, as well as by changing phosphor composition. Industrial white LED sources are commonly achieved by combining nitride-based single color LEDs with Y_3_Al_5_O_12_: Ce^3+^ (YAG: Ce) yellow emitting phosphor. This kind of white LEDs (WLEDs) used for illumination purposes are dominant in the market owing to their low energy consumption. YAG: Ce yellow phosphor-based white LEDs still suffer from significant limitations, such as low color quality and low spectral efficiency, which reduces their scope of application [38,39,40].

Optically pumped QDs were introduced as an innovative and promising class of phosphors in past few years. QD-incorporated LEDs (QD-LEDs) find their applications in a range of technologies such as general lighting, display backlights, and self-emissive displays. QDs have numerous potential advantages in terms of color quality, efficiency, and flexible incorporation into opto-electronic devices. The advantages of QDs as a color-converting material have been discussed in detail in the following section. By incorporating QDs as color-converter components, the output performance of LEDs can be significantly enhanced and by using the optical and electrical properties of QDs, a color-converted hybrid LED emitting at a longer wavelength can be developed. The process of color conversion with the incorporation of QDs is an equivalent method to the color conversion utilizing phosphors. However, the bandwidth emission for phosphor is very large compared to QDs that emit with the FWHM of around 40 nm which is significantly smaller than that of phosphors. In addition, a slightly smaller quantity of QDs may result in the process of color conversion, whereas very large quantities of phosphor are required to achieve full color conversion. Optical absorption is an important feature for the efficient operation of a hybrid color-converted system and hence the color-conversion materials should have decent absorption to exhibit high efficiency during the process of color conversion. QDs can absorb nearly all photons with wavelengths slightly shorter than those nanocrystals’ emission wavelength and hence these superlative qualities of QDs make them very promising candidates as an efficient color converter.

Two decades ago, one of the very first hybrid full-color LEDs based on GaN-LED and II-VI QDs was demonstrated by Lee et al. [41]. ZnS-coated CdSe and CdS QDs were pumped optically by means of GaN-based blue and ultraviolet LEDs to achieve emission wavelengths spanning almost the entire visible spectrum. In 2012, Zhang et al. [21] designed their own InGaN/GaN LEDs with a nanopillar structure and CdSe/CdS-based colloidal core/shell QDs to accomplish a hybrid light emitting device. QDs were mounted on the surface of nanopillar LED devices by dipping them in the QD solution, such that close proximity between the emissive quantum wells (QWs) and the QD layer was maintained. A strong non-radiative resonant energy transfer (NRET) from the QWs to QD layer was observed and described with the help of photoluminescence (PL) data. The internal quantum efficiency of nanopillar LED was reported to be enhanced by 263% in comparison with the planar LED owing to the non-radiative resonant energy transfer (NRET) process.

### 2.2. History of QD Patterning Technique

**Figure 1:**
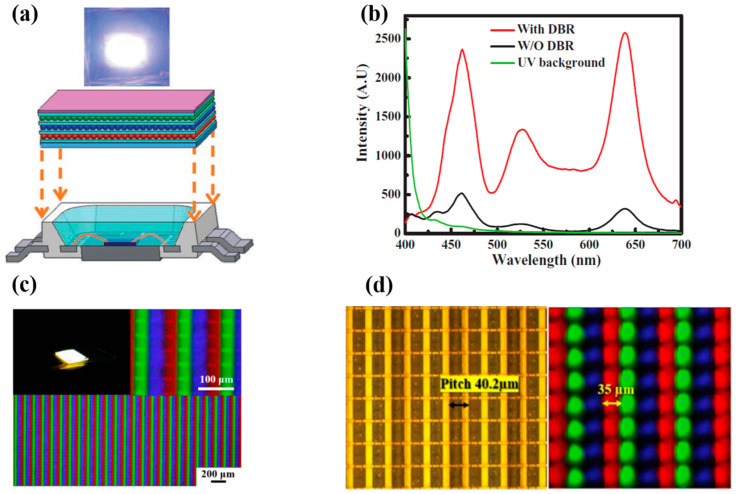
(**a**) Schematic of QD-LED device (bottom). Photograph of the QD-LED during operation (top). (**b**) EL spectra with and without the DBR structure. (**c**) Image of sprayed QDs μLED array under fluorescence microscopy at different magnifications. (**d**) Top-view image of the μLED layout with pitch of 40.2 μm defined as the intended channel (left). Quantum dots (QD) droplets jetted in the PR mold to confine the size and resolve the cross-talk effect (right) [44]. Figure reproduced with permission from John Wiley and Sons.

Displays are essential components of electronic systems and recent displays like micro-LED displays are based on small LED arrays. To integrate QDs into the array systems, we need to transfer QDs to a substrate surface and use it for display applications later on. To this end, several researchers implemented numerous techniques of QD printing or patterning, such as spray coating, aerosol jet printing, super-inkjet printing, etc. Hence, QDs are electrically and optically pumped to be used with light-emitting diodes. Colloidal QD-LEDs are used as color down-converters for light-emitting diodes to accomplish efficient illumination sources and high-quality displays. In 2012, Chen et al. [42] demonstrated QD-LEDs emitting all three primary colors by coating Cd-based colloidal QDs on a nitride-based UV LED. QDs were coated on the LED using a pulse spray method to ensure uniform deposition and fabricate large-area and small footprint QD-LEDs. A HfO_2_/SiO_2_ distributed Bragg reflector (DBR) was mounted on top of the device to ensure reusability of UV photons. A thin film of polydimethyl siloxane (PDMS) was deposited between individual layers with red, green, and blue emission to avoid cross-contamination. The large color gamut of these QD-LEDs makes them a suitable choice as back light units for display applications. Figure 1a shows the schematic of QD-LED and a photograph of the QD-LED during operation. Figure 1b shows the electroluminescence (EL) spectra of QD-LED with and without the DBR structure. Three emission peaks are observed at 460 530, and 640 nm. Further, the EL spectrum exhibits higher visible illumination upon adding the DBR structure, as the strong reflection of the UV band successfully suppresses the 395 nm peak and thus increases the intensity of visible light. Abe et al. [43] prepared a hybrid phosphor by mixing europium-doped strontium thiogallate (STG) green phosphor with red CdSe/CdS QDs and applied it on a blue LED to realize a display backlight. Therefore, this technique of pulsed spray coating is crucial to providing consistent RGB layers.

As mentioned earlier, QD-LED-based display technology is the ultimate choice for future generation displays because of their compelling potential advantages in terms of efficiency, power utilization, contrast ratio, lifetime, and response time. For a higher resolution, LEDs with a size smaller than 100 µm (known as µLEDs) must be used; hence, displays realized utilizing QDs and µLEDs are often referred to as QD-µLED displays. In 2015, Han et al. [45] reported a full-color display realized with the help of an ultra-violet (UV) µLED and colloidal QDs (CQDs). GaN-based UV µLEDs with a small pitch of 40 µm were combined with CQDs of all three primary colors, i.e., red, green, and blue. RGB QDs were deposited on the array of UV LEDs with the help of an aerosol jet (AJ) printer to ensure fine printing that is more precise and mask-less, and to enable non-contact deposition of liquids containing functional materials. The AJ system comprises two main parts; an ultrasonic atomizer and a spraying chamber. The QD suspension is atomized inside the atomizer using ultrasonic oscillations. The resulting aerosol is then transferred to the nozzle of the spray chamber using the flow of nitrogen gas. The nozzle of the AJ printer then prints QDs on the substrate with narrow linewidth and high resolution. A layer of DBR was used on the top of the device to enhance the utilization of UV light by reflecting leaked UV light back to the QD layers. The luminous flux of blue, green, and red colors was boosted by 194%, 173%, and 183% in comparison to the samples without DBR. The luminous efficacy of radiation was reported to be 165 lm/Watt under various currents. An QD- μLED array of 35 × 35 μm^2^ area comprised small emissive sections with a pitch size of 40 μm, as shown in Figure 1c. Each emissive section operated as a pixel with three sub-pixels emitting blue, green, and red colors. The device was further optimized in 2017, when Lin et al. [44] deposited photoresist (PR) mold between layers of each individual color to reduce optical cross-talk between them. This technique proved to be useful, as emission efficiencies of blue, red, and green QDs were reported to be enhanced by 5%, 23%, and 32%, respectively. PR mold was also found to be helpful in reducing the coffee-ring effect that leads to the non-uniform distribution of QDs on the LED surface shown in Figure 1d. This strategy is likely to be beneficial for future generation of light-emitting devices.

**Figure 2:**
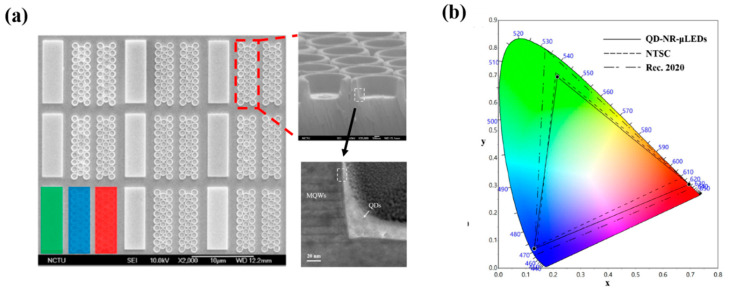
(**a**) Scanning electron microscopy (SEM) image of red, green, and blue (RGB) pixel array and NR-μLED with 30° tilt angle; transmission electron microscopy (TEM) image of the contact area between multi quantum wells (MQWs) and QDs. (**b**) Color gamut of RGB hybrid QD-NR-μLEDs, NTSC, and Rec. 2020 [46]. Figure reproduced with permission from Optical Society of America.

For a more better QDs printing technique than the previous technique, in a study by Huang Chen et al. in 2019, nanoring (NR) structures were fabricated on a green LED epitaxial wafer for the purpose of tuning the peak wavelength from green to blue through strain relaxation, in a method referred to as strain-induced engineering [46]. The epitaxial layers of GaN LEDs with 525 nm emission were grown on c-plane pattern sapphire substrates using metal organic chemical vapor deposition. The releasing strain technique was used to tune the emission wavelength from green to blue, and red QDs were deposited on the blue emission region for color down-conversion. Strain-induced engineering involves the reduction of LEDs volume by etching, which results in the release of strain and decrease in the quantum-confined Stark effect. This effect induces the flattening of the tilted energy band, expands the overlap among quantum state distributions, and blue-shifts the wavelengths. The final device comprises an array of RGB pixels with each containing a green subpixel, a blue subpixel with nanorings, and a red subpixel with nanorings as well as red QDs, as shown in the scanning electron microscopy (SEM) and transmission electron microscopy (TEM) images in Figure 2a. It is clearly observed from the SEM images that the sidewalls of the InGaN/GaN MQWs were closely surrounded by QDs which are important for the nonradiative resonant energy transfer (NRET) mechanisms. Alternatively, a 1-nm-thick Al_2_O_3_ layer deposited on the NR-μLED sidewall is obvious from the TEM image. The area of each subpixel was limited to 3 × 10 μm^2^ to maintain a sufficiently high resolution. For the deposition of a QDs in a tight space, the super inkjet (SIJ) printer was employed, which ensured the linewidth of the QD layer that is as thin as 1.65 μm. To passivate the sidewalls of the nanoring-μLED, a layer of Al_2_O_3_ was deposited through atomic layer deposition, which enhanced the PL intensity by 143.7% in comparison with the reference structure. The color gamut of the final QD-NR-μLED device was reported to be 104.8% and 78.2% according to NTSC and Rec. 2020 standards, respectively, as depicted in Figure 2b. Thus, we can precisely spray red QDs on a subpixel region of a blue NR-μLED using the SIJ printing strategy to ensure red-light emission.

**Figure 3:**
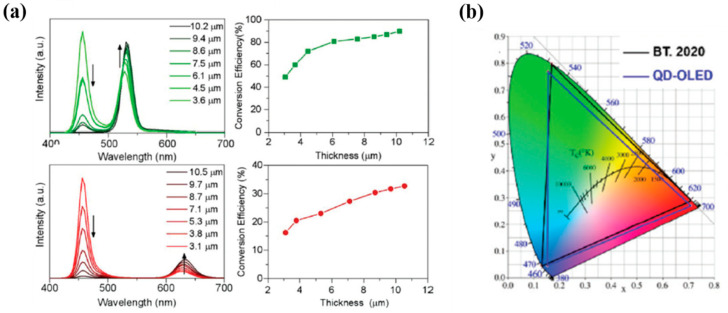
(**a**) Photoluminescence (PL) spectra and light conversion efficiency (LCE) of green and red QD layers at different thicknesses. (**b**) Color gamut of the QD-OLED display (blue triangle area), in comparison with the color gamut of BT. 2020 (black triangle area) [47]. Figure reproduced with permission from Royal Society of Chemistry.

Changing the thickness of micro-led is a must and can be configured by doing so to enable the color conversion mechanism from blue to green, or blue to red for full-color displays. Further study for inkjet printing has been done by Hu et al. by demonstrating uniform quantum dots printed in Inkjet as color conversion layers for full-color Organic LED displays in 2020 [47]. Figure 3a illustrates the measurements of produced a polymer-based QD ink with uniform green and red QDs at micrometer thickness. This indicates that the polymer-based QD inks effectively absorb blue light better than the solvent-based counterpart. The light conversion efficiency (LCE) from blue to green and red can be tuned by changing the thickness of the QD layer. The LCE of the green QD layer reached ∼90% at 10.2 μm thickness and ∼33% for red QDs at 10.5 μm thickness. The color gamut of the QD-OLED could reach ∼95% according to the BT 2020 standard, as shown in Figure 3b. This inkjet printing process introduced in this work offers a cost-effective way to expand QDs for full-color display applications.

**Figure 4:**
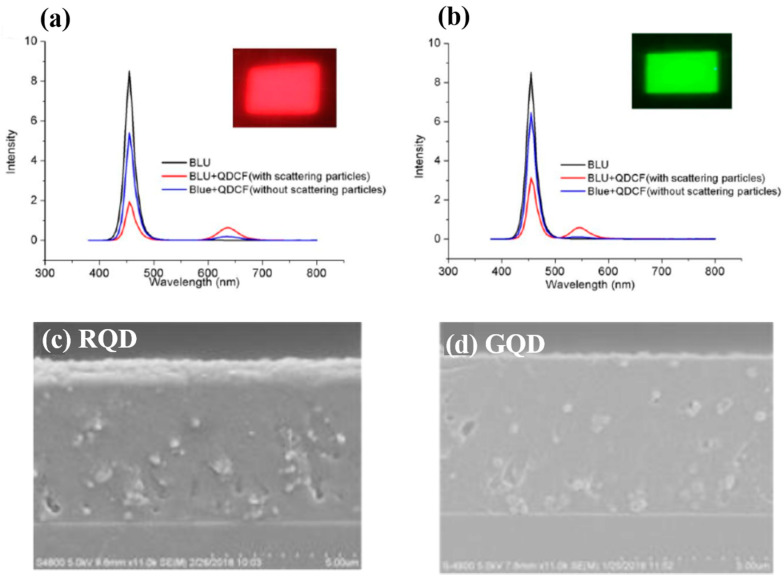
Comparison of (**a**) red and (**b**) green QDCFs spectra with and without scattering particles under blue LED backlight. (**c**,**d**) The cross-sectional SEM images of red and green QDPR on glass through photolithography process [48]. Figure reproduced with permission from John Wiley and Sons.

Despite the progress achieved by methods employed in previous articles, a facile and efficient technique for the patterning of QDs is needed for further development. Numerous researchers are currently studying QD deposition methods. The study of Yue et al. in 2018 [48] demonstrated the synthesis of QD-based photoresist (QDPR) with high stability and dispersity of QDs for the QDCF application, which achieved the fine-pitch by photolithographic patterning of QD embedded PR materials. Because of the composition of scattering particles, the absorption efficiency of QDCFs are obviously enhanced, as shown in Figure 4a,b. The absorbance of with and without scattering particles are 80% and 40% (QDPR-Red); the Green QDPR increases from 28% to 65%. Thus, the EQE of the Red and Green QDPR increases three and five times more than without scattering particles. Figure 4c,d show that the red and green QDs are efficiently dispersed in QDPR formulations.

**Figure 5:**
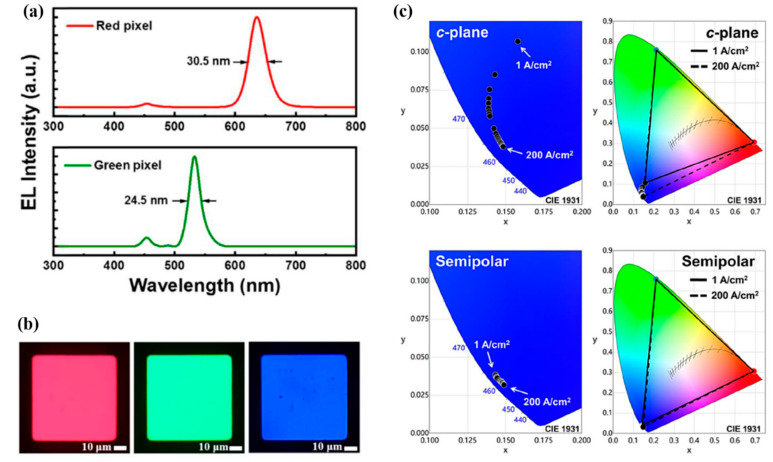
(**a**) EL spectra of red and green pixels. (**b**) EL image of RGB pixels. (**c**) Color gamut of RGB pixel assembly from c-plane μLED and QDPR at various current densities in CIE 1931 color space [49]. Figure reproduced with permission from Optical Society of America.

Further, the leakage of the blue incident light and cross-talk remain challenging issues for the μLED application. In 2020, Huang Chen et al. [49] combined the CdSe/CdZnS-based thick-shell QDs and black PR with TiO_2_, which can return the incident light to the QDPR and effectuate re-absorption in QDs. Black PR can absorb redundant incident blue light, as shown in Figure 5a. Hence, using the bottom side black PR and the upper side gray PR which provided height wall, the cross-talk between RGB pixels is prevented. The RGB EL microscope images are shown in Figure 5b. The use of the semi-polar μLED is another key point. Comparing c-plane and semi-polar μLED as sources, semi-polar devices exhibit a small wavelength shift during the driving current from 1 A/cm^2^ to 200 A/cm^2^. Figure 5c demonstrates the performance under the 1 A/cm^2^ to 200 A/cm^2^ driving current in the CIE 1931 colors space, and the coordinates varied from (0.1572, 0.1067) to (0.1483, 0.0379) and (0.1433, 0.0388) to (0.1490, 0.0317) for the c-plane and semi-polar blue μLEDs, respectively. Semi-polar devices demonstrate a wide color gamut of 114.4% NTSC and 85.4% Rec. 2020, which is attributed to the almost unchanged wavelength peak. A color conversion layer developed by QDPR is ideal for the typical lithography process and, consequently, for large-scale output.

**Figure 6:**
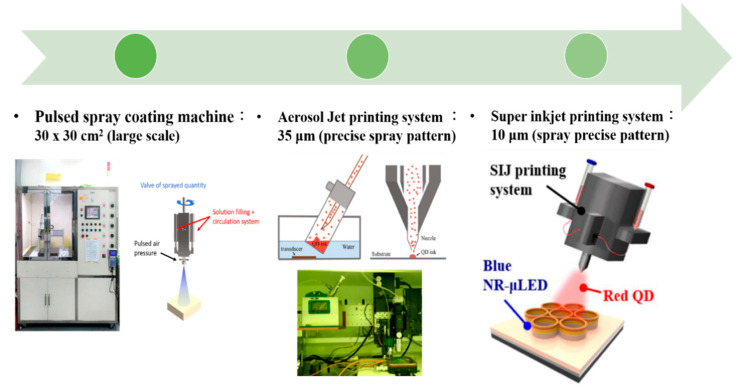
Developmental history of QD printing system at Kuo’s Lab [44,46]. Figure reproduced with permission from Optical Society of America.

In summary, the historical development of QD printing at Kuo’s Lab is illustrated in Figure 6. The overview of the developmental history of QD-based display technology with their description can be found in Table 1. First, the problem needs to be effectively solved. Thus, we will focus on fabricating the full-color display by improving every method. In the beginning, the spray coating machine was applied for large-scale chips. However, because of the non-uniform QD region obtained by this method, repeatability is challenging even under the same printing conditions. Moreover, the pulsed spray coating machine cannot control the precise linewidth. Subsequently, AJ printing was employed on the full-color LED, which combined PR mold and RGB QD pattern. The QD pattern layer is successfully controlled have thickness under 35 μm. In recent years, the super ink-jet printing (SIJ) technology appeared along with the widespread application of μLED. The SIJ can maintain the QD pattern layer below 10 μm, and it can further precisely jet the QD solution onto the surface. Moreover, QDs need to be printed repeatedly so that its layer becomes thick in order to avoid leakage of the blue incident light, which will take a lot of time. The method effectively uses QD-based photoresist to achieve fine pitch QD patterning. The advantage involves quick, thickness-controlled, large-scale processes which could possibly become the trend of the future. Most importantly, there are some issues that need to be resolved including the high blue incident light leakage, cross-talk effect, and so on. Hence the black matrix should prevent cross-talk effects because of its high contrast ratio. Additionally, the QDPR, which is a combination of QDs, photoresist, and TiO_2_ [49]. TiO_2_ can be considered a mirror to reflect incidents of blue light. This method can efficiently reduce outer blue light significantly. 

Further, a brief description about the developmental history of QD patterning technology is given in the following Table 1.

For a μLED, as the array size decreases, its light-emitting efficiency improves while experiencing massive non-radiative recombination from sidewall defects; however, the far-field radiation pattern will deviate from the ideal Lambertian distribution depending on the sidewall emission, resulting in the color shift of μLED displays. The color-conversion method and mass transfer process are two common solutions to achieving the full-color display; however, manufacturing challenges remain in terms of achieving high yield, large size displays such as tablets, monitors, TVs, video walls, etc., [50,51]. The most widely used commercial LED epitaxy wafer is based on structures of multi quantum wells (MQW), i.e., GaInP/AlGaInP MQWs for red LEDs and blue and green LEDs for InGaN/GaN MQWs [52], resulting in inconsistent angular distribution between RGB μLEDs due to different epitaxy materials and structures between them. Consequently, there is a color shift of mixed colors in RGB μLEDs displays.

**Figure 7:**
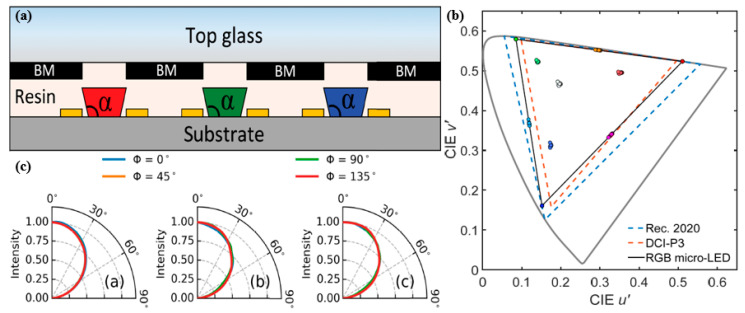
(**a**) RGB μLED display with top black matrix. (**b**) Simulated color shifts of ten reference colors at viewing angles from 0° to 80° for the RGB μLED display with a top black matrix and 120° taper angle. (**c**) Simulated radiation patterns for RGB μLEDs with top black matrix [53]. Figure reproduced with permission from Optical Society of America.

To minimize this particular color shift, Gou et al. introduced a device layout and studied the angular color shift of RGB μLED displays from a mismatched angular distribution suggesting a simulation model to support the experimental findings [53]. The author found that the green and blue chip sidewall emissions are far larger than those of red chips due to better absorption in red MQWs. The light emission from the red chip decreases because of the Lambert’s cosine law, while that of green and blue μLEDs increases and subsequently decreases. The difference in material dynamics of RGB chips will cause an angular shift in mixed colors. Owing to the low-cavity effect within μLEDs, no color change is observed at a fixed driving current for primary colors. The color shift problem is exacerbated as the size of the μLEDs decreases because of increased sidewall emissions from green and blue chips. To mitigate the color change, green and blue μLED sidewall emissions must be removed, and the RGB radiation patterns, i.e., the Lambertian distribution, must be obtained. Hence, Gou et al. suggested a system mode shown in Figure 7a, where the system consists of a μLED array and top black matrix outside the emission region. Further, the space between the μLEDs is filled with resin with a refractive index of about 1.5, such that the sidewall emissions from green and blue chips are completely absorbed by the black matrix, resulting in emissions from the top of RGB μLEDs with matched Lambertian distributions only. Moreover, the author introduces a taper angle, α, shown in Figure 7a, to boost the light intensity from the top emission. The light intensity from the green and blue chips is observed to increase as α increases from 90° to 140°, upon which it saturates, with little influence on the red chip. The color shift is extreme when α is larger than 130°, and the wider taper angle contributes to a narrower angular distribution between green and blue emissions, with no effect on the red channel, thus inducing a severe color shift. Figure 7b illustrates the simulated color shifts of ten reference colors at viewing angles from 0° to 80° for the RGB μLED display with top black matrix and 120° taper angle. The average color shift at 80° is 0.05, and the maximum value for the magenta channel is 0.014, which is below 0.02, and thus it is acceptable for commercial applications. The simulated radiation patterns for RGB μLEDs with the top black matrix clearly illustrate that the sidewall emission completely disappears owing to the presence of the black matrix, and the color shift is significantly reduced with a matched angular distribution for both RGB colors, as shown in Figure 7c. Therefore, with a high light extraction output, this device structure is believed to significantly suppress the color shift.

## 3. White-Light-Emitting Diode

Despite numerous challenges, such as sidewall emissions, as discussed above, LEDs have gained considerable attention over the past decade, both in the scientific community and in the industry, owing to their energy-saving capabilities [42,54,55,56]. White-light LEDs (WLEDs) have lower power consumption, high durability, smaller size, and high power efficiency compared to conventional lighting methods, hence they are expecting to become next generation solid-state lighting devices. In 1996, Nichia commercialized white LEDs by combining a blue LED chip with yellow Y_3_Al_5_O_12_:Ce^3+^ (YAG) phosphor. WLEDs were originally used for backlighting LCDs, but with improved output power, they will be increasingly used in flashlights and other lighting sources. Additionally, to enable the efficient use of white LEDs in general lighting, the luminous flux of a single white LED must be improved and to increase the luminous efficacy of white LEDs, the efficiency of blue LEDs needs to be increased, as they are the source of excitation light. Several studies have concentrated on improving the internal quantum efficiency (IQE) for the blue LEDs by enhancing the growth of crystals and researching to enhance the device’s light extraction efficiency.

Further, QDs are excellent materials for white LED (WLED) applications owing to their various basic properties, such as a wide absorption band, narrow emission peak, wavelength-dependent size which could cover all the visible spectrum by changing the particle size, high quantum yields (QYs), and good spectral overlap, all of which are the efficient improvement factors for WLEDs [57,58,59]. New forms of WLEDs have been developed over the past few years by combining CdSe-based QDs and yellow phosphor demonstrating a higher luminous efficacy compared to that using all phosphor materials, but such a structure leads to high QD reabsorption loss due to high thermal power and reduced optical stability [60]. Vertically layered packaging (VLP) structures are believed to enhance the optical efficiency of the QD-phosphorus (Q-P) hybrid WLEDs but unfortunately the backscattering issue of phosphor is not solved in such VLP structure [61,62,63,64].

### 3.1. CdSe QD-Based WLED

**Figure 8:**
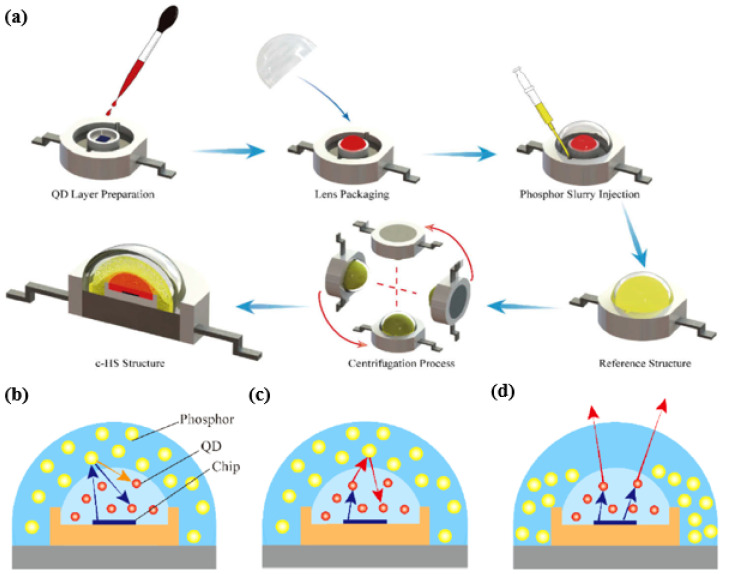
(**a**) Diagram of the fabrication process of WLEDs c-HS structure. Diagram of the light-extraction mechanism in Q–P hybrid WLEDs. (**b**) Backscattered blue light and backward-emission yellow light in the top region of reference structure; (**c**) backscattered red light in the top region of reference structure; (**d**) extraction red light with less backscattering in the top region of the c-HS structure [65]. Figure reproduced with permission from Optical Society of America.

Recently, Zongtao Li et al. has demonstrated a configuration-based quasi-horizontal separation (c-HS) structure to increase the QD light extraction efficiency of QD—phosphor-based white LEDs (WLEDs) to resolve this issue of backscattering problem [65]. This structural arrangement is reported to effectively suppress the backscattered loss from the phosphor at the top region of the QD layer giving excellent radiative power and luminous flux compared to the conventional vertical layered packaging structure. The fabrication process for this hybrid structure is shown in Figure 8.

**Figure 9:**
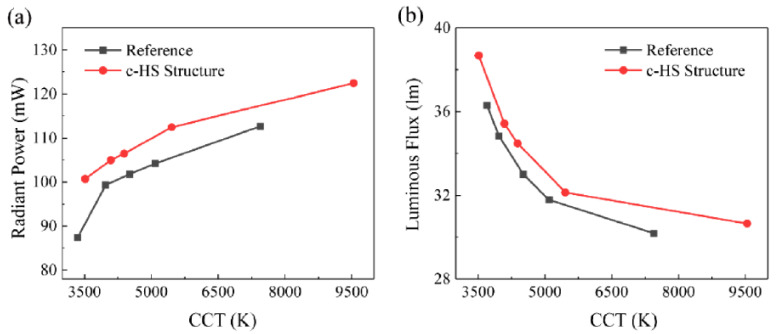
(**a**) Radiant power and (**b**) luminous flux of the reference structure and c-HS structure at different correlated color temperature (CCT) values [65]. Figure reproduced with permission from Optical Society of America.

In Figure 8a, QDs are mixed with silicone, then the mixture is dispensed on the blue chip and subsequently treated in the oven at 150 °C. A hollow semi-spherical lens is inserted into the frame of the device structure and phosphor slurry (phosphorus mixed with silicone) is then pumped into the hollow space followed by 3-hour heat treatment at 100 °C in the oven. Centrifugation technology is commonly used to monitor the phosphorus distribution in the device and this will help to increase the color uniformity of phosphor-converted LEDs [66,67]. Therefore, centrifugation technology is adopted for this c-HS structure to monitor the phosphorus settlement after injection of phosphor, which is again processed in the oven to complete the packaging. The author also discusses the effect of centrifugation speed on the optical performance of the device as the phosphor concentration is lowered on the top region of the structure with increase in centrifugation speed. Because of the strong scattering effect of phosphor, the backscattered blue light emitted from the blue chip and the backward emission light from the phosphor will penetrate through the QD layer again and increase QD’s absorption efficiency, thus emitting more red light from QD. Thus, with an increase in centrifugation speed, the concentration of phosphorus decreases in the top region of the device, then the backscattering probability of red light from phosphor decreases, suppressing the portion of red light that penetrates through the QD layer thus reducing the reabsorption loss which can be seen from Figure 8b–d. In this way, more red light of QDs can be extracted from the device as the centrifugation speed increases. In addition, the radiant power and the luminous flux for the reference and c-HS structure are compared with the various correlated color temperature (CCT) values as shown in Figure 9a,b and from the figure it is very clear that the radiant power and the luminous flux of the Q-P hybrid WLEDs with c-HS structure are obviously higher than that of the reference structure with an improvement of 13.6% and 10.8% respectively, at a standard warm white color of ~4000 K. The c-HS structure is therefore beneficial for less backscattered red light and better QD-layer light-extraction performance.

Commercial WLEDs are generally produced using the blue GaN LEDs to pump yellow phosphor, as discussed in the above section. But under high current injection, GaN-based LEDs confronted some efficiency droop problems like current droop (reduction of efficiency with increase in current) and thermal droop (reduction of efficiency with increase in temperature) [68,69]. Some researchers have identified the cause for this efficiency droop, and it is due to various factors such as built-in fields [70], Auger recombination [71], and electron leakage [72]. Some researchers have suggested in recent developments that these limiting factors can be reduced by employing InGaN QDs for LEDs in the active layer. Chunyu Zhao et al. has demonstrated a green light emitting InGaN QD LED structure grown on c-plane sapphire substrate using metal organic chemical vapor deposition (MOCVD) technique showing a reduced efficiency droop, high temperature stability, and strong internal quantum efficiency (IQE) for the LED operating in “green gap” [73]. Green gap refers to the reduction of efficiency of LEDs in the green-yellow range of the visible spectrum. The QD LED structure consists of GaN capping that protects the QDs during subsequent GaN growth at high temperature.

**Figure 10:**
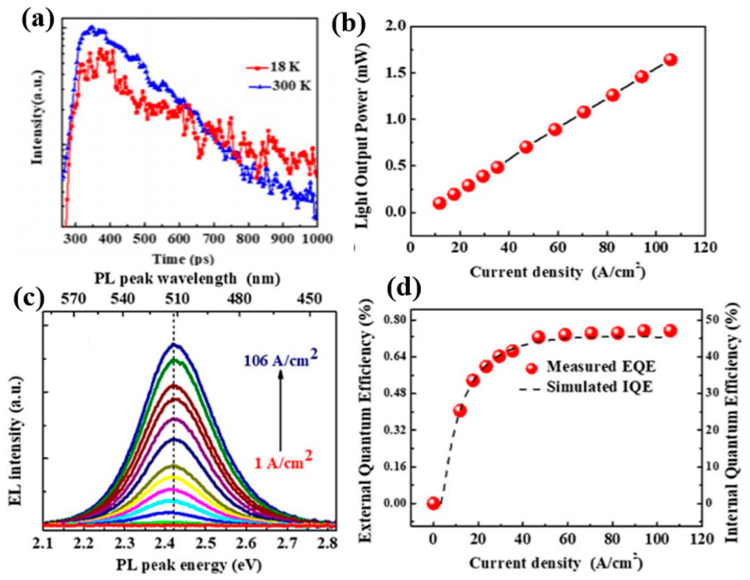
(**a**) TRPL measurements of capped QDs; (**b**) LOP versus injection current density; (**c**) electroluminescence spectra of QD LED; (**d**) EQE as a function of current density [73]. Figure reproduced with permission from Optical Society of America.

Figure 10a represents the TRPL measurement showing the mono-exponential decay spectrum of the capped QDs at 300K and 18K. The lifetime is achieved at 480 ps at 300 and the radiative lifetime at 18 K is 707 ps indicating that non-radiative recombination at low temperature is fully suppressed. Since the radiative lifetime is inversely proportional to the overlap of electron and hole wave functions, this shorter lifetime is a solid indication of built-in fields reduction. The light output power (LOP) of the device is shown in Figure 10b showing a linear increase with respect to the injected current density up to 106 A/cm^2^. To investigate the built-in field in the QDs, the electroluminescence (EL) spectra of the LED is measured for different injection currents at room temperature which are shown in Figure 10c. It is apparent that there is no shift of EL peak wavelength as the injection current is gradually increased from 1 A/cm^2^ to 106 A/cm^2^ suggesting a fully screened built-in fields and yet green QD LEDs with no observed efficiency drop have not yet been identified. Figure 10d depicts the external quantum efficiency (EQE) of the LED as a function of the injection current density and it is very clear that the efficiency without droop up to an injection current density of 106 A/cm^2^ is due to fully screened built-in field thereby inducing an increase in the rate of radiative recombination. The Auger recombination coefficient is calculated from the theoretical simulation of IQE as shown in the figure and found to have a very low value which indicates that it does not contribute to the efficiency droop. Hence, there is strong evidence that InGaN QDs can substantially enhance LED performance and solve efficiency droop problems with fully screened built-in fields.

### 3.2. Perovskite QD-Based WLED

**Figure 11:**
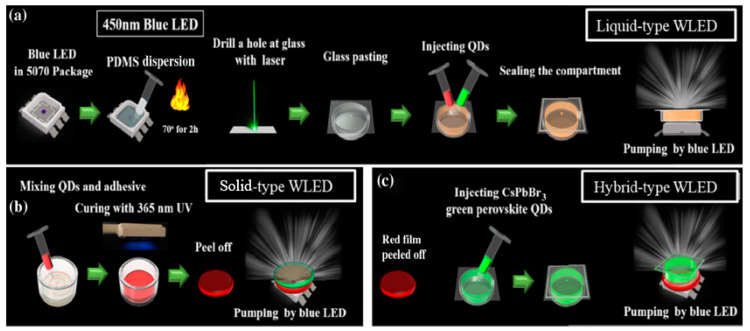
Process flow of perovskite quantum dots (PQDs)-based WLEDs: (**a**) liquid, (**b**) solid, and (**c**) hybrid types [74]. Figure reproduced with permission from Optical Society of America.

In the recent years, halide PQDs were demonstrated as spectacular semiconductors for lighting applications. As stated in the previous sections, PQDs offer significant advantages in terms of photoluminescence quantum yield, high color purity, more tolerant to defects, etc., compared to other types of QDs, hence they are promising for next-generation display and lighting technologies. Lin et al. demonstrated hybrid-type white LEDs based on inorganic halide PQDs in three different geometries including liquid, solid, and hybrid types; they presented performance improvements for realistic applications by addressing the issues mentioned above [74]. Figure 11a–c shows the systematic procedure for the fabrication of liquid-, solid-, and hybrid-type device structures.

**Figure 12:**
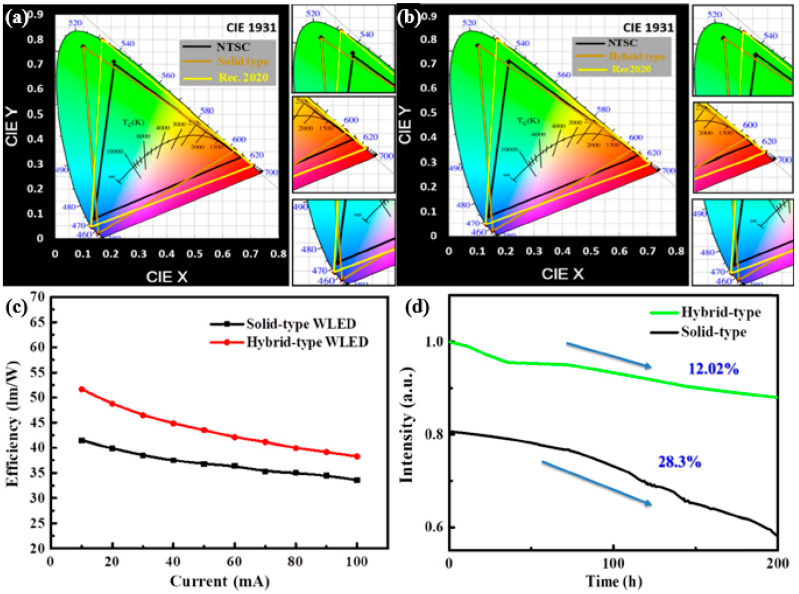
Color gamut on CIE 1931 color space for (**a**) solid and (**b**) hybrid-type WLED. (**c**) Luminance efficiency with respect to current. (**d**) Stability of solid- and hybrid-type WLEDs [74]. Figure reproduced with permission from Optical Society of America.

In this study, hot-injection methods were employed to synthesize the inorganic green (CsPbBr_3_) and red (CsPbBr_1.2_I_1.8_) light halide PQDs, all of which were pumped by the blue LED. Figure 12a encompasses a wide area of NTSC (120%) and Rec. 2020 standards (90%), while Figure 12b covers the wider areas of 122% of NTSC and 91% of Rec. 2020 standards. Although the solid-type structure system exhibited large values in the color gamut, it was not as powerful as the hybrid-type. Hence, the decrease in productivity depends primarily on the type of packaging [75]. Aggregated solid-type QDs enclosed in silicone resin can cause dispersion and re-absorption, leading to loss of efficiency [58,76], and it is believed that the efficiency of QDs may be improved by changing the structure and dispersion of QDs. Consequently, the hybrid type exhibits improved performance and a wide color gamut compared to both the liquid and solid forms.

The emission spectra appear unchanged after 200 h of aging, leading to a nearly constant area of color gamut because of constant spectral form. The stability of the fabricated devices can be estimated from Figure 11. The performance based on the current-dependent efficiency for both solid- and hybrid-type WLED is shown in Figure 12c, indicating that the efficiency for the hybrid-type is 51 lm/W, whereas that for the solid-type is 41 lm/W, demonstrating a significant improvement in case of the hybrid-type. The self-aggregation of QDs in solid PQD films is the main cause behind the efficiency loss [77]. Figure 12d illustrates the luminance efficiency of the devices, showing a decrease of only 12.02% for the hybrid-type WLEDs and a corresponding decrease of 28% for the solid-type after being operated for 200 h. From these results, it is inferred that the hybrid-type device exhibits a substantial increase in stability, aside from a high performance compared to the solid type. Moreover, the temperature in the solid-type device was found to be 78 °C at 250 mA. This high surface temperature arises from the non-radiative relaxation energy. Consequently, the system suffers from a decline in performance and reliability. In contrast, the hybrid-type unit has a low surface temperature, i.e., 40 °C at 250 mA, which makes the system more reliable in terms of performance and quality. The hybrid structure is being proposed to solve the red perovskite anion exchange problem to achieve an ultra-high gamut; however, the reliability of this design must be further improved to meet specific application requirements in future research. Several attempts have been made over the past decade to develop highly functional and long-lasting solutions for the use of WLEDs’ application in display technology and room lighting systems.

**Figure 13:**
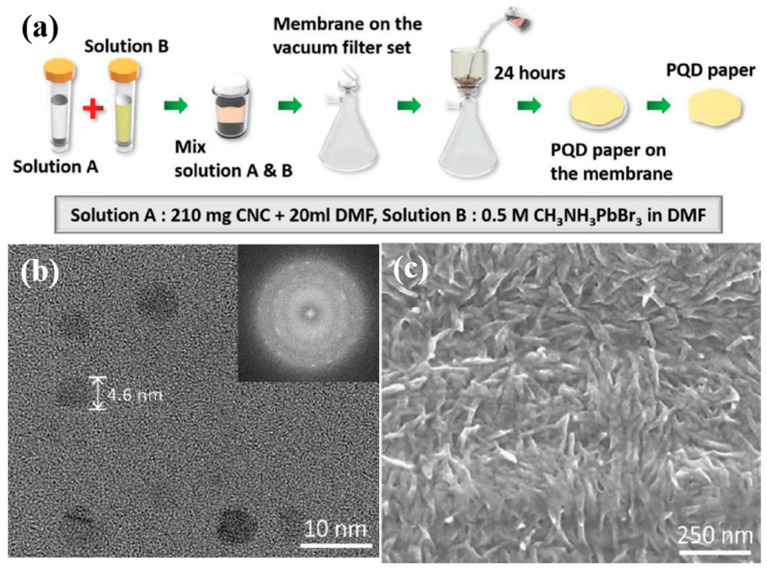
(**a**) Schematic of fabrication process of PQD paper. (**b**) TEM image of CH_3_NH_3_PbBr_3_ PQDs. (**c**) SEM of PQD paper surface [78]. Figure reproduced with permission from John Wiley and Sons.

In another research for PQDs-based, Kang et al. demonstrated white light emitting diodes (WLEDs) using PQD paper exhibiting an ultrahigh luminous intensity, wide color gamut, and long operational lifetime [78]. The aim of this research was to establish a cost-effective and scalable production technique to resolve high-energy radiation by demonstrating a paper manufacturing process using cellulose nanocrystals (CNCs) to manufacture a new form of PQD film. Figure 13a illustrates the manufacturing process of PQD paper. The CNC suspension and CH_3_NH_3_PbBr_3_ are combined in dimethylformamide, upon which the mixture is dried on the membrane to produce the PQDs paper. In comparison to other approaches, this technique is fast, convenient, and consumes less time to aid in the rapid development of PQD paper. The inclusion of cellulose nanocrystals (CNC) enables the orderly arrangement of the crystal structure, thus creating mechanical strength in the paper and allowing the capping ligands to encapsulate the growth of the QD structures by reprecipitation, thereby contributing to strong fluorescence emission. However, the use of CNC degrades the quantum yield of the PQDs, as the CNCs absorb light in the ultraviolet region. From the TEM and SEM images shown in Figure 13b,c, it is evident that the size of the PQDs lies within the range of 3–8 nm, which facilitates a powerful quantum confinement effect. This confinement effect plays a major role in enhancing the light emitting efficiency of the perovskite.

**Figure 14:**
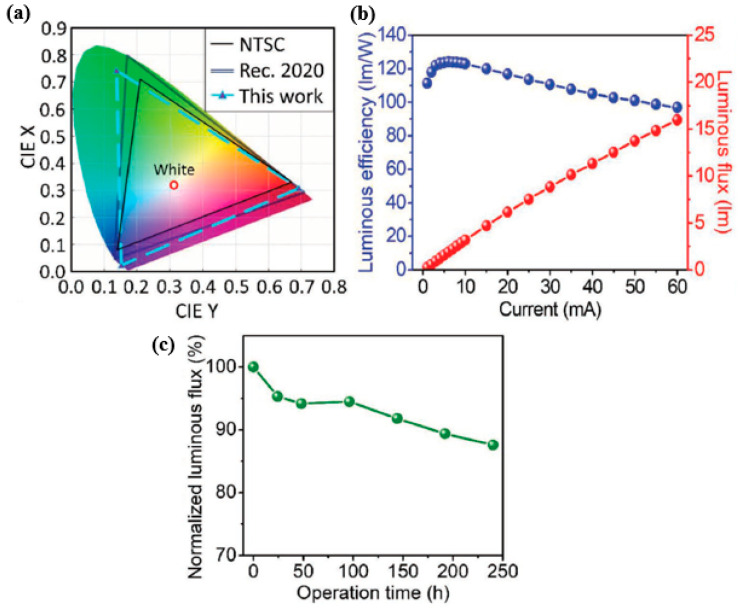
(**a**) CIE diagram illustrating the color gamut of the NTSC standard, the Rec. 2020 standard, and the PQD paper-based LED. (**b**) Current-dependent luminous efficiency and luminous flux of the PQD paper-based LED. (**c**) Time-dependent luminous flux of the LED device under continuous operation [78]. Figure reproduced with permission from John Wiley and Sons.

The emission peak also corresponds to the absorption edge, and the PQDs paper can serve as a light converter for the blue GaN-LED chips, as it exhibits high absorption characteristics in the short wavelength region. In this study, the authors focused on using PQDs paper as green converter for white LED, which is achieved by placing the PQDs paper on top of the blue LED package dispensed with a mixture of red K_2_SiF_6_: Mn^4+^ (KSF) phosphor and silicon resin. The PQDs paper-based LED displays a wide color gamut of 123% according to the NTSC standard and 92% according to the Rec. 2020 standard, as shown in Figure 14a. This is mainly suitable for 8K4K display technologies of the next decade [79]. Figure 14b depicts the current-dependent luminous efficiency of the PQD paper-based LED, showing a high value of 124 lm/W at 6 mA. Even at different drive currents, the PQD paper-based LED exhibits high performance by consistently exhibiting a luminous efficiency above 100 lm/W. Further, the reliability of the device is depicted by the red line in Figure 14c, indicating only 12.4% depletion of the luminous flux after 240 h of operation.

**Figure 15:**
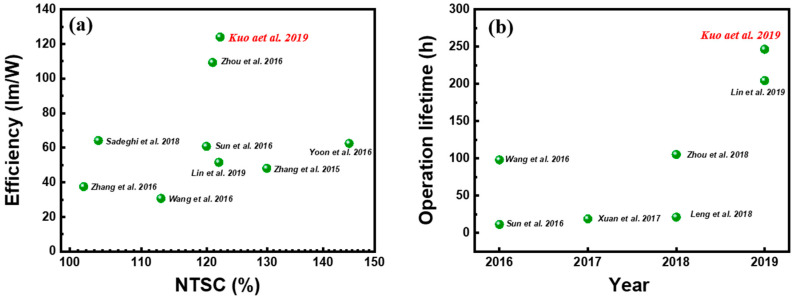
Comparison of device performance between PQD paper-based LED and other reported LEDs using QDs as color converters. (**a**) Luminous efficiency and color gamut performance and (**b**) operational durability of reported QD-based LEDs [78]. Figure reproduced with permission from John Wiley and Sons.

Figure 15a,b provides a summary of the PQD paper-based device and other reported LEDs using QDs as the color filter. The main reason behind the high performance of this PQD paper-based LED is attributed to the QD structure structured inside the cellulose material, which is primarily because capping ligands play a major role in creating a strong complex structure on the PQD surface, thereby enhancing the stability of the PQD [32]. Some of the challenges faced by numerous researchers are the loss of ligands, as for conventional colloidal PQD synthesis, the long chain oleic acid (OA) and oleylamine (OLA) ligands are not tightly bound to the PQD surface and can be lost during the manufacturing process, making them the key factor for PQD instability [80]. In this paper, the issue of ligand loss is completely avoided, as the PQDs produced are in solid form, as the growth of perovskite is accompanied by the CNC capping ligands, leading to well-capped and stable PQDs. Hence, the developed PQD paper demonstrates an impressive ability to produce high-efficiency white LEDs with a wide range of colors, indicating a promising display technology material.

**Figure 16:**
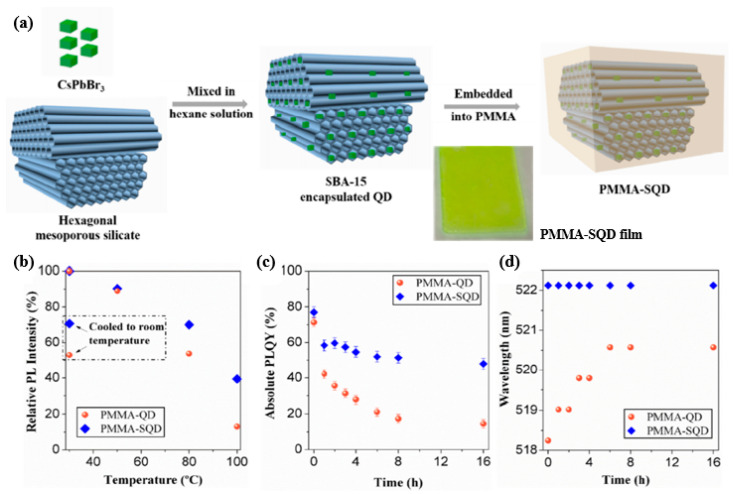
(**a**) A schematic diagram for the preparation process of PMMA-SQD composites; (**b**) the relative PL intensity as a function of heating temperature from 30 °C to 100 °C; (**c**) the PLQY values and (**d**) PL peak wavelength as a function of time when the films continuously heated at 80 °C [82]. Figure reproduced with permission from Optical Society of America.

In practical applications, however, PQDs face difficulties, such as instability, anion-exchange reactions, QD surface oxidation, photo-bleaching, and aggregation [24,81]. As mentioned in the previous section, encapsulation is one of the methods used to increase the stability of PQDs and with this Yujun Xie et al. demonstrated encapsulated room-temperature synthesized CsPbX_3_ perovskite quantum dots for display applications showing high stability and wide color gamut to solve these problems [82]. The fabricated green-emitting and red-emitting QDs are being used for the application of quantum dot-converted white LEDs showing a wide color gamut of 135% NTSC and 101% Rec. 2020 respectively. First, green emitting CsPbBr_3_ and red emitting CsPbBr_1.2_I_1.8_ QDs are synthesized based on the method of supersaturated recrystallization. The fabricated QDs are mixed with mesoporous silicates (SBA-15) in hexane solution and then stirred 600 rpm for 1 h followed by centrifugation at 4000 rpm to obtain the nanocomposite powder of SBA-15 encapsulated QDs after drying. In addition, the encapsulated QDs obtained were dispersed in poly (methyl methacrylate) (PMMA) and the preparation process is shown in Figure 16a and subsequently the PMMA-SQD composite films were prepared by casting on a quartz substrate. The PMMA-QD composite films were also prepared for references. Figure 16b evaluates the thermal stability of the composite films, which shows that the PMMA-SQD film is more resistant to the thermal effect as it shows a better recovery of relative PL intensity rising back to 70.7% when the temperature is cooled down from 100 °C to room temperature. The measured photoluminescence quantum yield (PLQY) value for PMMA-SQD and PMMA-QD films are respectively 77% and 71.4% but after heat treatment for 16 h, the PMMA-SQD film still has a PLQY value of 48% without any peak wavelength shift while the PMMA-QD film has only 14.4% and the peak was red-shifted by more than 2 nm as shown in Figure 16c,d.

**Figure 17:**
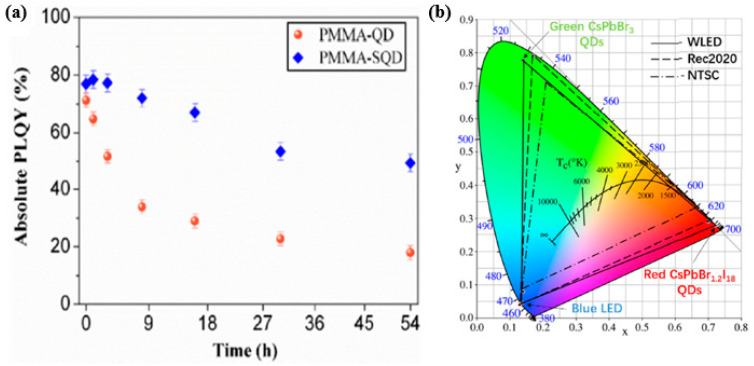
(**a**) The PLQY values under UV irradiation; (**b**) color gamut of the resulting WLED [82]. Figure reproduced with permission from Optical Society of America.

The photo stability analysis of the composite films is evaluated for the films with continuous UV irradiation at a wavelength of 365 nm and from Figure 17a, it is clear that the PLQY of the PMMA-SQD films decreased from 77% to 48.5% after 54 h continuous irradiation, while the PMMA-QD films has only 18% PLQY. To demonstrate the display application for the perovskite composite films, WLED is being created by mounting the green and red emitting QDs films on a blue-LED chip. The color gamut of the resulting white LED is measured and it is able to achieve a wide color gamut of 135% NTSC and 101% Rec. 2020 as shown in Figure 17b. The combination of PMMA and SQD thus leads to outstanding performance of the device by suppressing unfavorable grain growth and surface trap sites.

### 3.3. Flexible WLED

**Figure 18:**
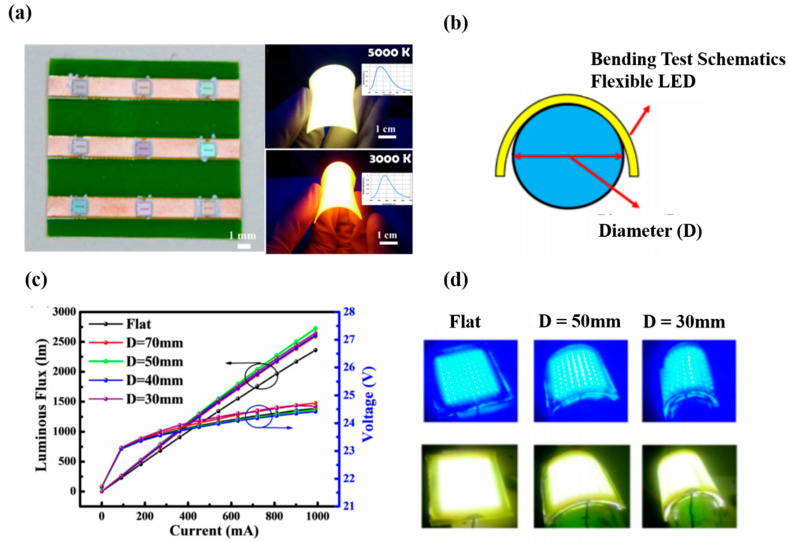
(**a**) LED array under optical microscope (left), phosphor layers at 5000 K and 3000 K (right top and bottom, respectively). (**b**) Schematic of flexible device. (**c**) Voltage and luminous flux with respect to current at different bending diameters for the flexible white LED. (**d**) Top and bottom photographs depict before and after bonding the adhesive phosphor film and blue LED arrays, respectively [85]. Figure reproduced with permission from Optical Society of America.

The WLEDs discussed above are typically brittle and hard, as the bonding strength between the QDs powders and the substrate material is inadequate, often resulting in reduced substrate extensibility for flexible display devices. The development of a stretchable and tough white-light emitting material for flexible display devices is therefore very important. Rogers et al. extensively investigated flexible devices and used transfer printing technology to achieve the goals of flexible lighting technology [83]. However, the technology proved to be too complicated to manufacture the devices. The commonly applied method is to combine the solid-state lighting and curved light guide; nevertheless, the curved light guide is so stiff that it is unsuitable to be bent numerous times, as required for flexible devices [84]. In 2015, Kuo et al. [85] used the flip-chip, silicone-based anisotropic conductive adhesive, and phosphor film, which easily achieved the fabrication of a flexible lighting device. A uniform flexible LED, high-efficiency illumination, and a wide range of bending was achieved without deterioration of the total light output. Figure 18a shows an optical microscope image of the LED array, and Figure 18b shows the flexible LEDs operating under different bending curvatures. The size of this flexible white LED is ~5 × 5 cm^2^, and the total thickness is approximately 6 mm at the cross-section of the flexible white LED structure. Figure 18c shows the voltage and luminous efficiency as a function of the current, indicating little variation up to a bending diameter of 3 cm. Figure 18d shows the top and bottom pictures before and after bonding of the adhesive phosphor film and blue LED arrays, respectively. The results indicate that this large area flexible LED array is suitable for flexible displays and devices.

A brief description and comparative study of different types of displays based on QDs, PQDs, and flexible one have been made in the following Table 2 showing their performance and some of the challenges they are facing. Therefore, the future of display technology is quite bright with the advent of QDs in this field and many exciting and unpredictable things can be expected to happen in the coming years with the expectation that everyone can get a highly efficient, reliable, and low-power display technology.

## 4. Conclusions

This study reviews the trends in QDs-based display technology, mainly focusing on μLEDs, including array structures and other perovskite QDs-based LEDs that exhibit high efficiencies and high polarization features. Owing to their exciting potential benefits in terms of performance, power consumption, contrast ratio, lifetime, and response time, QD-LED-based display technology is considered the ultimate option for future generation displays. The development of QD printing technology has been described, including methods of pulsed spray coating, aerosol jet printing, and super inkjet printing. We moreover discuss PQDs-based white light LEDs exhibiting ultra-high luminous intensity and long operation lifetimes. Several challenges facing PQDs, such as heat, pressure, pressure, high-energy radiation, etc., were overcome using various methods developed by researchers that are explained in this study. Moreover, colloidal QD-LEDs are used as color down-converters for LEDs to achieve effective sources of illumination and displays of high quality. A breakthrough in the field of QDs-based μLEDs for use in display technology is certainly expected in the near future.

## Figures and Tables

**Table 1:** Development in QD patterning technology.

	Methods	Description
**Pulsed-Spray Coating Machine**	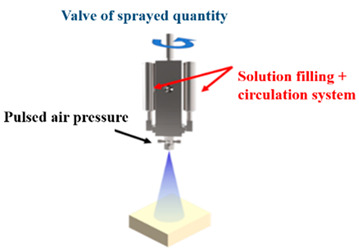	The operation is precisely controlled by a computer and its quality of spray was quite stable each time. The disadvantage is the spray diameters which is large, which could not be used to spray precise pattern.
**Aerosol Jet Printing System**	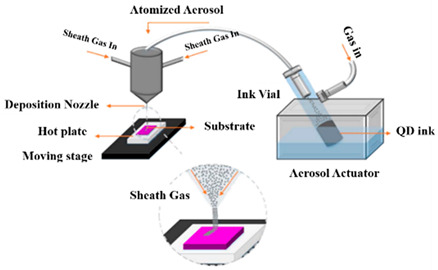	The QDs solution was aerosolized by the ultrasonic vibration. The Aerosol Jet process began with a mist generator that atomized liquid materials into small droplets. The disadvantage is the large spray area, and QDs also printed out of the windows.
**Super-Inkjet Printing System** [46]	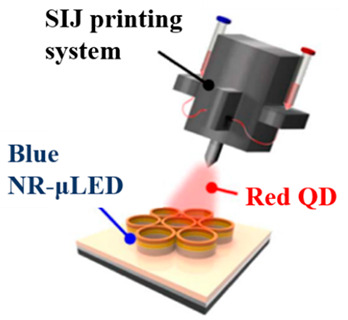	The pressure generated by the oscillating electric field is used by this printing machine to print the QDs. With better control of QDs-inks, it provides fine-linewidth pattern. In addition, printing sufficiently dense QDs caused the higher color conversion of the color Red and Green is time consuming.
**Quantum Dots Photoresist methods**	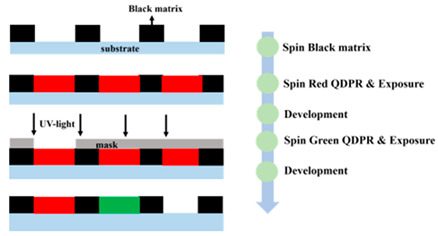	Quantum dots photoresist methods by photolithography is a fast and convenience method. This method can control the thickness of QDPR to prevent the leakage blue light. However, the disadvantage is QD usage is high.

**Table 2:** Comparative study of different display types.

Types	Performance	Challenges
**QDs-based displays (CdSe)**	Improved color and energy efficiency, good at absorption high energy blue light and re-emitting at longer wavelength. CdSe-based QDs has more than 90% PLQY and less than 30 nm FWHM leading to better color quality.	Reduction in EQE of the devices, subjected to non- recombination processes including surface trapping, Auger recombination, etc. Toxicity of Cd-based QDs limits their use.
**PQDs-based displays**	Impressive ultra-narrow linewidth of 15–18 nm for green, tunable wavelengths leading to higher efficiency and brightness. Emission of light strongly and efficiently in a variety of colors.	Stability issues making them susceptible to degradation from high temperature and light flux. Color shift problem. Content of lead in PQDs.
**QDs-based Flexible displays**	Growing demand for flexible and wearable displays, integration of flexible QLEDs with wearable sensors, micro-controllers, wireless communication units for next generation consumer electronics.	Some of the issues include thinness and robustness of the display, reliability, high cost, poor optical clarity, issue of mass production
